# Improving palliative care outcomes in remote and rural areas of LMICs through family caregivers: lessons from Kazakhstan

**DOI:** 10.3389/fpubh.2023.1186107

**Published:** 2023-08-03

**Authors:** Islam Salikhanov, Maria C. Katapodi, Gulnara Kunirova, Byron L. Crape

**Affiliations:** ^1^Department of Clinical Research, University of Basel, Basel, Switzerland; ^2^President of the Kazakhstan Association of Palliative Care, Almaty, Kazakhstan; ^3^Nazarbayev University School of Medicine, Nur-Sultan, Kazakhstan

**Keywords:** palliative care, stakeholders, challenges, family caregivers, LMICs

## Abstract

Approximately 60 million people require palliative care worldwide, and nearly 80% of them live in low- and middle-income countries (LMICs). Providing palliative care in remote and rural areas of LMICs requires special consideration to ensure equitable access to healthcare. This perspective aims to deliver pragmatic, context-oriented policy recommendations designed to improve palliative care outcomes in Kazakhstan by capitalizing on existing resources and considering its unique geopolitical and sociocultural context. With approximately half of the population in Kazakhstan residing in remote and rural regions, the provision of healthcare services – specifically palliative care – mandates particular attention to ensure equal access to high-quality care. To understand challenges of implementing palliative care in remote and rural regions of Kazakhstan and to propose tailored solutions, 29 key stakeholders, including family caregivers, health professionals, and palliative care administrators, were identified in five regions of Kazakhstan. The main challenges encountered by family caregivers include lack of palliative care skills, the need for home-based care from mobile services, and high out-of-pocket expenditures. The challenges highlighted by healthcare providers and administrators were the lack of formal education in palliative care, shortage of opioids, and limited societal awareness and state support. Based on challenges elaborated from stakeholders and existing literature in palliative care and family caregiving, this perspective advocates against replicating the strategies implemented in high-income countries. Family caregivers play a critical role in implementing affordable and efficient palliative care in resource-limited settings. Enhancing their competencies through digital training and increasing access to palliative care services through mobile teams are tailored and localized solutions that address specific challenges in Kazakhstan. It is postulated that these recommendations may find utility in other LMICs, potentially benefiting nearly 48 million individuals who require these services.

## Introduction

WHO defines palliative care as an approach that enhances quality of life of patients and their families facing life-threatening illnesses (1). It aims to alleviate suffering through early identification, comprehensive assessment, and pain management, while also addressing physical, psychosocial, and spiritual problems (1). This care philosophy affirms life, accepting dying as a normal process, providing support for an active life until death, and extending support to the family throughout and after the patient’s illness (1). Approximately 60 million people need palliative care worldwide in 2020, and nearly 80% of them live in low- and middle-income countries (LMICs) (2–4). Despite growing demand, access to palliative care in LMICs remains limited. The demand for palliative care in LMICs is projected to double by 2060, yet these countries lack the necessary infrastructure to establish and distribute these services, especially in rural and remote areas (4, 5). According to the United Nations, 3 billion people worldwide who live in rural and remote areas face significant challenges such as poverty and limited access to healthcare and education, creating critical challenges for policymakers and development organizations (6). The Lancet Commission on the Value of Death suggested that strengthening palliative care services in LMICs requires comprehensive approaches that consider the unique challenges faced by communities in these settings and leverage innovative solutions to improve access to care (3).

Investing in palliative care in LMICs has the potential to improve health equity worldwide (2, 3). WHO estimates that only 14% of patients worldwide who need palliative care have access to such services, primarily in countries with more robust economies (7). This leads to an unequal distribution of suffering among patients and their families, especially among those who are economically disadvantaged, socially excluded, or reside in remote and rural regions. Palliative care in LMICs can improve the quality of life of patients and their family caregivers by increasing access to medications essential for pain and symptom management (8, 9). Studies in Kenya, India, and Bangladesh found that introducing palliative care services in rural districts led to long-term cost-savings, as patients received home- and community-based care, reducing the need for costly hospitalizations and other healthcare services (4, 10). Palliative care services can contribute to health equity by addressing the underlying social determinants of health, such as poverty, lack of access to services, and discrimination (6, 11). As the demand for palliative care in LMICs increases, it is important to identify tailored local solutions. Adopting the strategies and approaches of high-income countries is neither feasible nor sustainable due to limited resources and lack of healthcare infrastructure in LMICs.

## Knowledge GAP

There has been significant attention dedicated to the challenges faced by palliative care patients in remote regions. These challenges are well-documented in the literature, as evident in 30 systematic reviews published with the last 9 years, which emphasize the need to develop healthcare solutions tailored to LMICs (8, 12–38). However, there is significant lack of recommendations for the development of context-specific and tailored solutions suitable and sustainable for resource-limited nations (2, 3, 39). Despite the fact that the majority of palliative care patients in LMICs reside in remote rural areas, the literature lacks recommendations on how to address the distinct challenges they face within their unique cultural, economic, financial, and national contexts in LMICs (2, 3, 19).

The Lancet Commission on the Value of Death underscores the significant role of community healthcare workers in providing palliative care in remote and rural regions. However, even though this approach has demonstrated its effectiveness in higher income settings, it may not necessarily be feasible in LMICs due to shortages of workforce, funding, and infrastructure (2, 3, 22). Hence, there is a need to generate research-based insights that can foster the development of recommendations uniquely adapted to the conditions of these rural and remote settings, thereby, better serving the majority of palliative care patients in LMICs (2, 3, 22, 40). Therefore, this perspective aims to deliver pragmatic, context-oriented policy recommendations designed to improve palliative care outcomes in Kazakhstan by capitalizing on existing resources and considering its unique geopolitical and sociocultural context. While the Lancet Commission provides a broader framework for the development of healthcare solutions, this perspective provides context-specific, tailored recommendations that are solidly grounded in the challenges reported by stakeholders in resource-limited settings of Kazakhstan (2, 3).

## Palliative care in Kazakhstan

Situated in Central Asia, Kazakhstan is a low-middle income country characterized by a unique geography that significantly impacts healthcare delivery. Spread across 2.7 million km^2^ with a sparse population of only seven individuals per km^2^, Kazakhstan’s vast and disperse demographic landscape presents considerable challenges to accessing health services, especially for the nine million inhabitants residing in remote and rural areas (41).^1^ This problem is notably acute in palliative care delivery, a burgeoning need fueled by the country’s demographic shift towards an increasingly aging population – a trend consistent with other LMICs (42). Despite an estimated 107,000 individuals currently requiring palliative care services, the resources remain scarce (4, 43). With only 45 physicians and 101 nurses serving 1,925 palliative care beds, the Quality of Death Index places Kazakhstan 50th out of 80 countries (44). As reported by the World Hospice Palliative Care Alliance, Kazakhstan’s palliative care system is only at a preliminary stage of integration into the healthcare system, indicating a pressing need for development to meet national requirements and international standards (4, 43). Since 2016, Kazakhstan implements ‘The Road Map of Palliative Care Development,’ a strategy outlining key steps for policy development, educational initiatives, and service implementation, all tailored to enhance palliative care services uniquely suited to the country’s context (45).

This perspective examines the challenges of developing palliative care services in Kazakhstan, as an example of developing such services in LMICs that are searching for affordable solutions to transform their own healthcare system. According to the Lancet Commission Report, lack of data hinders the evaluation of palliative care services in LMICs (3). Addressing this challenge requires research on stakeholder needs, i.e., family caregivers of terminal patients, healthcare providers, and policy makers (46, 47). This Perspective presents a comprehensive synthesis of challenges faced by key stakeholders in palliative care in Kazakhstan, and offers suggestions for improving palliative care outcomes in resource-limited and remote and rural settings. Our suggestions could be relevant to other LMICs in Central Asia beyond Kazakhstan.

## Challenges of palliative care stakeholders in Kazakhstan

We identified 29 key stakeholders in palliative care in Kazakhstan between August 2021 and April 2022, and assessed their needs regarding palliative care services, along with the challenges they encountered. Supplementary Table S1 provides a comprehensive overview of the demographic characteristics of our diverse group of stakeholders, which includes family caregivers, physicians, nurses, and administrators. Stakeholders were identified from three hospices and three cancer centers located in five different regions of Kazakhstan, spanning the northern, southern, and eastern parts of the country. Only one cancer center and two hospices were located in the major cities of Astana and Almaty, respectively. We assessed their needs and challenges through semi-structured interviews conducted in Russian (Supplementary Table S2). All participants were fluent in the language. Following data collection, we utilized a descriptive content analysis approach to identify the key challenges faced by palliative care stakeholders. Supplementary Table S3 contains representative quotes from participants across all stakeholder groups, illustrating the key insights that emerged during the interviews. The procedures adhered to the good research practice guidelines of the Medical Research Council (50). The Nazarbayev University Institutional Research Ethics Committee (IREC413/24052021) approved the study.

The key stakeholders comprised 12 adult family caregivers, 12 healthcare providers, and 5 administrators of palliative care services. Family caregivers assisted with palliative procedures (such as massage, hygiene, prevention of bedsores, etc.) for terminally ill cancer patients who had been receiving inpatient palliative care for at least 14 days. The healthcare providers, consisting of five physicians, five nurses, and two psychologists, each had a minimum of 3 years of experience in palliative care. Both family caregivers and healthcare providers were recruited from the same facilities. The administrators, who were employed by Non-Governmental Organizations (NGOs), frequently interacted with the Ministry of Health and participated in policymaking, each having a minimum of 5 years of experience in palliative care services.

**Figure 1:**
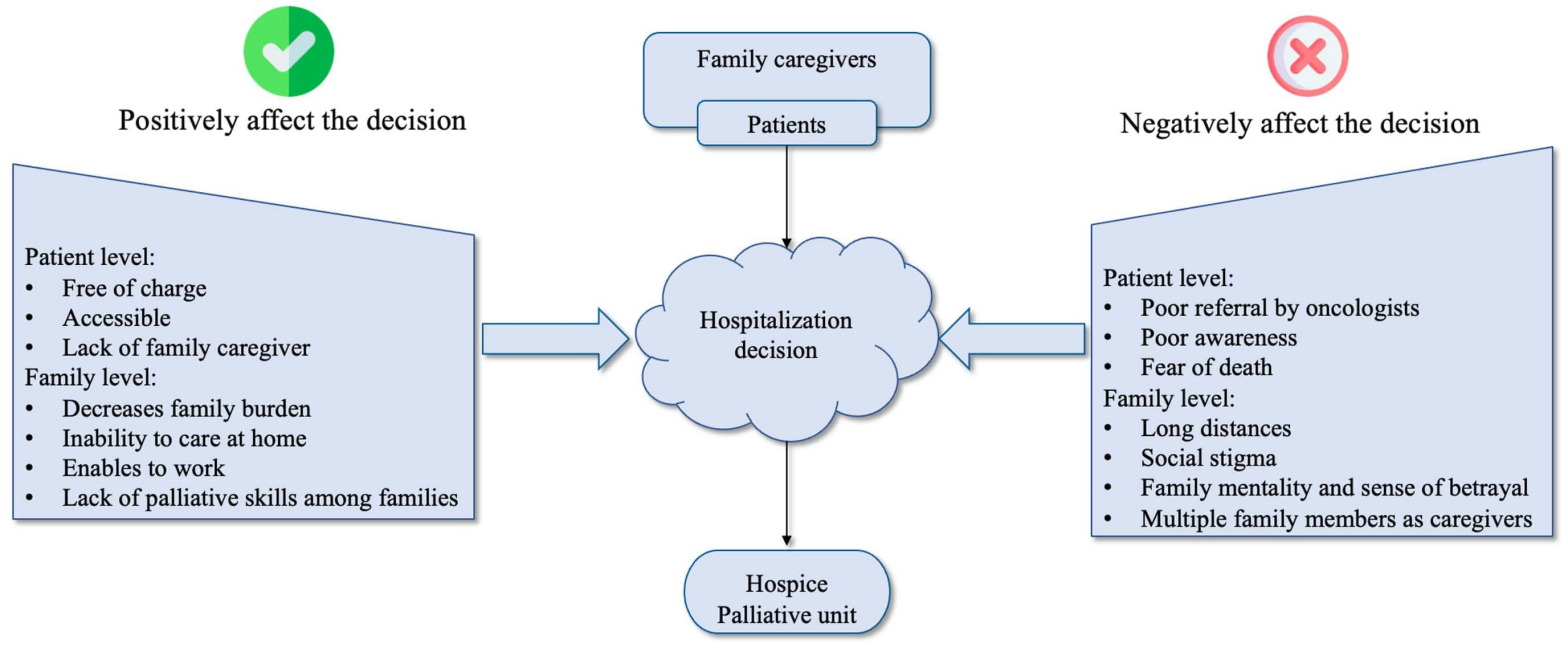
Factors associated with the decision to receive inpatient palliative care.

Figure 1 summarizes the factors influencing family caregivers’ perceptions of palliative care, reflecting their understanding of the patients’ experiences and perceptions. This figure presents the challenges reported by family caregivers and further elaborated on by healthcare providers and administrators. The goal is to demonstrate that future policies and interventions should be tailored to address the factors negatively affecting perceptions about palliative care, such as inadequate referrals from healthcare providers. Reflecting on the roadmap for palliative care development in Kazakhstan, we outline specific challenges to providing palliative care in the country and suggest recommendations to address these issues (48).

### Lack of caregiving skills, mobile palliative care services for home-based care, and loss of income and high out-of-pocket expenditures

Palliative care services in Kazakhstan, encompassing both inpatient and outpatient settings, are heavily dependent on family caregivers. This is particularly the case in remote and rural areas where half of the population resides. However, most family caregivers lack the knowledge and practical skills related to patient care. Although nursing staff provide demonstrations of basic procedures in inpatient settings, these are very unsystematic. A terminal cancer diagnosis and the subsequent necessity for family caregiving often result in a significant loss of income for the entire family, either due to the patient’s inability to work or the family caregiver leaving their job to provide care. Most family caregivers favor home-based care and support from mobile teams over inpatient palliative care services. Home-based care would enable many of them, especially in remote and rural areas, to minimize long-distance travel to inpatient services, retain jobs, and minimize the loss of income for the entire family. However, mobile teams are largely unavailable, e.g., there is only one mobile team that covers the palliative care needs of Almaty, a city with a population of two million.

The lack of sufficient state funding and universal health coverage results in high out-of-pocket medical expenses, which consume a large portion of family income. This financial burden further exacerbates catastrophic health expenditures for families living in remote and rural areas and increases inequalities, with some families being able to afford more expensive treatment, equipment, and consumables than others.

### Poor formal education and shortage of opioids for pain management

The formal education of healthcare providers in palliative care is insufficient due to a shortage of academics and other teaching staff with expertise in this field. Only a small number of physicians and other healthcare providers have received training abroad through state-funded educational programs. The majority of training is acquired ‘on the job’, which often results in a poor understanding of the nature of palliative care. This issue is particularly impactful on the nursing workforce, where heavy workloads and unmet expectations can lead to burnout and a high turnover rate among new nurses. Remote and rural areas suffer disproportionately from these issues because trained specialists typically prefer to seek employment in larger cities rather than rural areas (2, 49).

The lack of formal education of healthcare providers in palliative care often leads to a fear of prescribing opioids (opiophobia) among physicians and oncologists (50, 51). In Kazakhstan, 95% of terminally ill patients suffer from severe pain at the end of their lives and do not have access to opioids (48). The problem has been exacerbated by increased government control in attempt to combat drug trafficking. Few medications are available for pain control, including only weak opioids and small amounts of oral morphine, making access to pain medication difficult in remote and rural areas. This leads to many avoidable hospitalizations as patients are forced to be admitted to a hospice or palliative unit to receive opioids.

### Lack of societal awareness and state support

A general lack of awareness about palliative care within the broader population presents another barrier to the development and delivery of effective services. Misunderstandings about the role of palliative care often create false expectations that patients will receive curative treatment. These unmet expectations can lead to stigmatization of palliative care services and foster anger and hostility towards healthcare providers. This issue is exacerbated in remote and rural areas where healthcare awareness is generally lower. The absence of robust and comprehensive policies and regulations regarding palliative care in Kazakhstan has led to the development of these services without active governmental involvement. As a result, palliative care often remains unincorporated into existing healthcare systems, leading to issues such as a lack of accountability, insufficient quality control, and limited availability and accessibility of palliative services (7). Stakeholders suggest that the key to further developing palliative care involves enhancing cooperation between stakeholders and the government, as well as garnering increased support from governmental organizations.

## Recommendations

**Figure 2:**
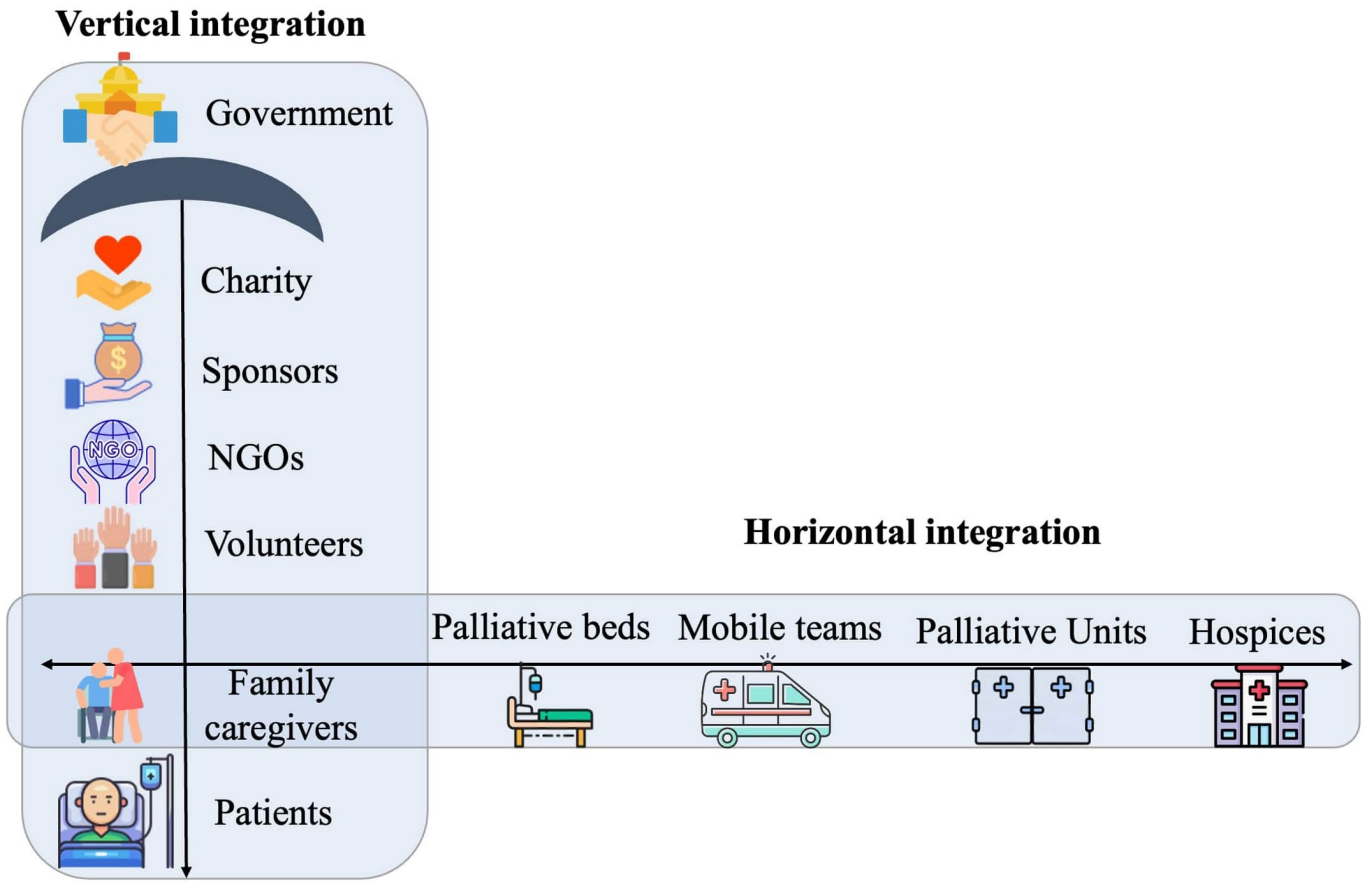
Vertical and horizontal integration of palliative care services.

Palliative care in Kazakhstan is currently delivered in various settings, such as hospices, palliative units of cancer centers, sparse mobile teams, and single palliative beds in general hospitals (48). The understanding of, and approach to, palliative care varies greatly across these settings. To address the establishment and expansion of palliative care in Kazakhstan, both horizontal and vertical integration of the existing diverse services should be implemented (Figure 2).

Horizontal integration aims for the standardization and consistency of palliative care delivery across different settings such as mobile teams, hospices, and palliative care units. This would include a uniform approach to medical procedures, pain and symptom management, medication availability, spiritual patient support, and comprehensive family caregiver assistance, which encompasses skills training, psychosocial support, and grief counseling.

For instance, out of 1,900 palliative beds in the country, 1,100 are single beds scattered across various units (e.g., general therapy, pulmonology) and dispersed over a vast geographical area. Data on these single palliative beds is scarce, making it challenging to understand the differences and effectiveness of these services. Implementing horizontal integration could involve redistributing these single beds into newly established hospices or increasing the number of mobile units, with the aim of achieving a consistent understanding and practice of palliative care across all settings. The focus should be on creating or maintaining a homelike environment with significant family caregiver involvement.

Vertical integration, on the other hand, refers to a hierarchical structure of palliative care stakeholders and parties involved, with the government taking the leading role in policymaking (as shown on p.37 of the Global Atlas of Palliative Care) (4). The government would form a policy umbrella over other parties involved, such as NGOs, charities, and volunteers. Vertical integration could enable smoother policymaking and more clearly defined roles in palliative care management and decision making. In Kazakhstan’s current palliative care scenario these roles are not well defined, while NGOs often assume a leading role in palliative care policy and funding. The absence of the Ministry of Health as a major stakeholder is a limitation of this perspective, considering its crucial role as a primary healthcare stakeholder with responsibilities in shaping policies and distributing resources.

### Enhance competencies of family caregivers through training and increase access to palliative care through home-based mobile services

Improving family caregivers’ competencies through training and expanding access to palliative care via home-based mobile services can be both financially feasible and cost-effective, especially in remote and rural areas of Kazakhstan and potentially other LMICs. Home-based care not only reduces costs compared to inpatient care but also enhances patient outcomes by increasing access to essential care and reducing hospitalizations (52). Increasing the number of mobile teams would also help address disparities in accessing these services in remote and rural areas, where traditional healthcare facilities may be limited or non-existent (9, 40, 52). Given that the majority of palliative patients in Kazakhstan are cared for by their families, equipping family caregivers with proper training can support them in delivering high-quality home-based care. This approach not only lessens the burden and financial strain associated with terminal disease but also ensures optimal use of scarce resources at both family and societal levels (53). Training programs could focus on enhancing caregivers’ knowledge and self-efficacy in basic palliative care procedures, such as hygiene and feeding, while also offering resources to support them psychologically and address caregiver burden (54, 55). Guided by mobile teams, trained caregivers will be more capable of managing pain, preventing bedsores, and addressing other symptoms. Leveraging the surge of digital technologies in the post-Covid-19 era, online and m-Health courses could reach family caregivers even in remote and rural areas of Kazakhstan and other LMICs (56, 57).

The literature extensively emphasizes the importance of family caregiver training and support, particularly in LMICs (3, 53–56, 58–62). Family caregivers are acknowledged as integral to long-term care, and all health professionals are encouraged to incorporate them into care teams and provide enhanced support to families (63).^2^ The growing body of evidence underscores the need to address the challenges faced by family caregivers in these contexts, thus reinforcing our recommendations for enhancing competencies and implementing comprehensive programs for family caregivers in Kazakhstan (3). The sense of coherence, rituals, traditions, and long-term mutual support that families and communities provide to the dying or grieving cannot be replaced by healthcare professionals (3). Education platforms for family caregivers have already demonstrated the feasibility of achieving significant enhancement in the well-being of patients and their families (64, 65). In rural and remote areas, family caregivers struggle with inadequate healthcare infrastructure and, even more, with a shortage of qualified healthcare personnel. Therefore, they should be given particular attention in the context of palliative training (66). In 2017, it was estimated that in the US alone, 41 million family caregivers provided 34 billion hours of care, corresponding to an economic value of $470 billion (63). Given these estimates and that palliative care relies heavily on family caregivers in Kazakhstan, our recommendation for supporting and training them becomes imperative for LMICs.

### Establish a comprehensive palliative care system and increase awareness of palliative care in remote and rural areas

Increased opportunities to educate healthcare providers and access to pain medication are interconnected key components of the horizontal integration of palliative services, promoting a consistent approach to care delivery in different contexts of remote and rural regions (67). Training in palliative care would help minimize variations in care provision, enabling uniform approach to effective use of medication for pain management, symptom control, and psychosocial support across all settings. By integrating pain management into the horizontal axis of palliative care services, healthcare providers can ensure that patients receive the care they need regardless of their geographic location or socioeconomic status. This entails streamlining bureaucratic procedures related to the import and distribution of pain medications to reduce delays and ensure their availability.

Local production of pain medications could lower costs and lessen the disproportionate economic impact of fluctuating exchange rates on LMICs. The successful implementation of strategies for affordable local morphine production in Uganda in 2003 exemplifies the importance of promoting local production of pain medication. This strategy significantly improves the quality of life of patients and family caregivers and remains affordable for LMICs (68).^3^ In Uganda, the cost of 110 days of pain management with oral morphine equals the price of a loaf of bread, thereby providing essential pain relief and ensuring a satisfactory quality of life for all palliative patients until death (68). The Kazakhstan Association of Palliative Care successfully engaged the Ministry of Health and the Police Department to facilitate a five-fold increase in the availability of fentanyl patches. This accomplishment underscores the importance of advocacy and collaboration in addressing the country’s palliative care needs. Annual awareness-raising campaigns organized by the Kazakhstan Association of Palliative Care, supported by hospices, hospital units, physician organizations, and NGOs, attract hundreds of volunteers and generate considerable social media attention. These campaigns advocate for an integrated approach to palliative care, emphasizing its long-term societal benefits (69).^4^

## Discussion

Our recommendations, based on the unique challenges and needs of LMICs, prioritize the efficient use of available resources. In this perspective, we argue against adopting a universal approach that merely replicates the expensive strategies of high-income countries, as it is neither sustainable nor advisable. Instead, we endorse the adoption of more nuanced, tailored, and context-specific approaches. Some specific practices, interventions, and policies prevalent in high-income countries (HICs) might be adaptable or translatable for palliative care interventions in LMICs. These may include:

**Low-cost medications**: some HICs use expensive medications for symptom management. In LMICs, affordable, generic, and essential medications should be prioritized, and alternative treatments that are more accessible should be explored.

**Nonspecialized workforce**: HICs often have a specialized workforce dedicated to palliative care. In LMICs, training non-specialist healthcare providers such as primary care providers and nurses in palliative care principles may be more feasible and sustainable, enabling them to provide care within their communities with the assistance of family caregivers.

**Basic infrastructure**: HICs may have specialized facilities for end-of-life care. In LMICs, integrating palliative care services into existing hospices or developing home-based services may be a more feasible approach, especially with the assistance of family caregivers.

**Integrated care systems**: some HICs have multiple uncoordinated palliative care providers. In LMICs, it is vital to develop a coordinated, collaborative approach that engages all stakeholders in optimizing resources and ensuring continuity of care. Creating a centralized system that connects healthcare providers, NGOs, and government agencies can help coordinate and optimize resources and ensure more efficient care provision.

Replicating strategies of HICs could lead to the misallocation of scarce resources and the introduction of policies that do not resonate with local populations’ needs, thereby hindering the development of palliative care services in LMICs (70, 71). The insights gained from the current advancements in palliative care in Kazakhstan present invaluable lessons about the challenges and opportunities inherent in developing such services in other LMICs. These insights highlight the importance of crafting local solutions to cater to the unique needs of these populations, with family caregivers as an integral part of these solutions.

Target 3.8 of the United Nations Sustainable Development Goals underlines the objective of attaining universal health coverage by 2030, which includes access to crucial health services and protection from financial risks (72).^5^ However, the realization of universal health coverage is unattainable without palliative care. Despite the evidence-based nature of this perspective, we intentionally focused on formulating tailored policy recommendations in response to the well-documented challenges. By highlighting these key challenges and recommendations, this perspective can provide guidance to health authorities and policymakers in LMICs striving to improve palliative care within their communities. A shift towards community-based care can reduce healthcare costs, improve patients’ access to care – especially those who might otherwise lack it – and enhance the overall well-being and quality of life of remote and rural communities in LMICs.

## Data availability statement

The raw data supporting the conclusions of this article will be made available by the authors, without undue reservation.

## Ethics statement

The studies involving human participants were reviewed and approved by Nazarbayev University Institutional Research Ethics Committee (IREC413/24052021). The patients/participants provided their written informed consent to participate in this study.

## Author contributions

IS conceived and designed the analysis, collected the data, analyzed the data, and wrote the paper. MK conceived and designed the analysis, contributed data and analysis tools, and wrote the paper. GK collected the data and wrote the paper. BC conceived and designed the analysis and wrote the paper. All authors contributed to the article and approved the submitted version.

## Funding

This project has received funding from the European Union’s Horizon 2020 research and innovation programme under grant agreement No 801076 for IS, and Swiss Cancer League KLS-4294-08-2017, PI: MK for the CASCADE study.

## Conflict of interest

The authors declare that the research was conducted in the absence of any commercial or financial relationships that could be construed as a potential conflict of interest.

## Publisher’s note

All claims expressed in this article are solely those of the authors and do not necessarily represent those of their affiliated organizations, or those of the publisher, the editors and the reviewers. Any product that may be evaluated in this article, or claim that may be made by its manufacturer, is not guaranteed or endorsed by the publisher.

## Footnotes

^1^https://data.worldbank.org/indicator/EN.POP.DNST?end=2020&locations=KZ&start=1961&view=chart

^2^http://resource.nlm.nih.gov/101767885

^3^https://www.hospice-africa.org/uganda/

^4^https://www.thewhpca.org/events-2021/item/1750-meters-above-sea-level-whpcday21-in-almaty

^5^https://unstats.un.org/sdgs/metadata/?Text=&Goal=3&Target=3.8
