# Supplementary material for: Improving palliative care outcomes in remote and rural areas of LMICs through family caregivers: lessons from Kazakhstan
Source: Front Public Health. 2023 Aug 3;11:1186107. doi: 10.3389/fpubh.2023.1186107 (PMC10434554; doi:10.3389/fpubh.2023.1186107)
Supplement: Supplementary file 1 [file Table_1.DOCX]

|  |  | **Family Caregivers** | **Health professionals** | **Administrators** |
| --- | --- | --- | --- | --- |
|  | Category | % (n) or Median (Range) | | |
| **N** |  | 41% (12) | 41% (12) | 18% (5) |
| **Gender** | Female | 92% (11) | 92% (11) | 60% (3) |
|  | Male | 8% (1) | 8% (1) | 40% (2) |
| **Age (years)** |  | 45 (24–58) | 50 (23–59) | 49 (38-56) |
| **Settings** | Hospice | 58% (7) | 58% (7) |  |
|  | Cancer Center | 42% (5) | 42% (5) |  |
| **Specialty** | Physician |  | 42% (5) |  |
|  | Nurse |  | 42% (5) |  |
|  | Psychologist |  | 17% (2) |  |
|  | NGO |  |  | 60% (3) |
|  | Health consulting |  |  | 20% (1) |
|  | Palliative facility |  |  | 20% (1) |
| **Experience (years)** |  |  | 19 (3-30) | 10 (9-25) |
| **Relationship to patient** | Wife | 17% (2) |  |  |
|  | Daughter | 58% (7) |  |  |
|  | Mother | 8% (1) |  |  |
|  | Niece | 8% (1) |  |  |
|  | Son | 8% (1) |  |  |
| **Type of cancer** | Breast | 33% (4) |  |  |
|  | Bowel | 25% (3) |  |  |
|  | Lung | 25% (3) |  |  |
|  | Uterus | 8% (1) |  |  |
|  | Other type | 8% (1) |  |  |
| **Length of time since diagnosis of cancer** | 1–≤ 3 months | 17% (2) |  |  |
|  | 3–≤ 6 months | 8% (1) |  |  |
|  | 6–≤12 months | 17% (2) |  |  |
|  | >12 months | 58% (7) |  |  |

**Supplementary Table 1.** Characteristics of study participants
