# Supplementary material for: Improving palliative care outcomes in remote and rural areas of LMICs through family caregivers: lessons from Kazakhstan
Source: Front Public Health. 2023 Aug 3;11:1186107. doi: 10.3389/fpubh.2023.1186107 (PMC10434554; doi:10.3389/fpubh.2023.1186107)
Supplement: Supplementary file 2 [file Table_2.DOCX]

**Supplementary table 2**

**Interview guides for Family caregivers**

|  | **Theme** | **Aim** | **Questions / probes** |
| --- | --- | --- | --- |
| **1** | Start of the caregiving process | Rapport building.  Understand initial experience with palliative care. | 1. What relationship do you have with the person, to whom you provide care to?      1. Tell me about one of your days, starting from the morning, for example on Monday...  - What kind of care do you conduct on daily basis? - How much time do you spend on these caregiving activities on a typical day? - Do you have someone from your family/friends who helps you with caregiving on a regular or irregular basis? Who is this person?  1. Do your remember when it started? At what occasion and how it happened?  - When did you start to provide the care - How the state of health of “name” has changed since then? - What help are you able to provide easily? - What help is hard for you to provide? Why is it so?  1. How was it decided that you are the one in your family taking over these tasks? |
| **2** | Identifying work-life balance of family caregiver | Discover the burden of family caregiving | 1. How do you balance providing care and your personal life?  - Which feelings do you experience providing care? - Do you feel stress? If yes how do you deal with it ? - How much time do you dedicate to your work, leisure time, and sleep while being a caregiver? - Has it changed since becoming a caregiver? How?  1. Do you communicate with others about your current situation as a caregiver of “name”? 2. What do you tell them? Can you speak to somebody if you have difficulties?    - Do others enquire about your wellbeing? Who? 3. What would make you stop providing care to your relative? |
| **3** | Identifying facilitators and difficulties | Identify difficulties that influence caregiving process and strategies for improvements | 1. Going back to the care you are now giving, what are the difficulties?  - What should be improved according to you?  1. What can people from your family and social network do to help you to improve the situation?  - How your relationships with your relatives and friends changed since you became a caregiver?  1. How the care affects your financial condition?  - How much money do you spend out-of-pocket? |
| **4** | Relationships between family caregiver and patient | Identify perceived effects and outcomes of caregiving and coordination | 1. How do you think your presence affects the care?  - How relationships with the patient changed after becoming the caregiver? - Who do you think should contribute more to the care: hospice staff or family? |
| **5** | Communication with the hospice staff | Assess the quality of communication between staff and family caregivers | 1. How would you assess your relationships with hospice doctors/nurses that treat “name”?  - Can you describe the quality of communication with hospice staff? - How do they help and support you? - Are you satisfied with the hospice care?  1. How do you seek information when you need help?  - Are you satisfied with the given information?  1. Have you been taught providing palliative care?  - If yes, What kind of teaching or training you received? |
| **6** | Closing questions | Views and opinions | 1. Is there anything else you would like to share on this topic? |

**Interview guide for Physicians**

|  | **Theme** | **Aim** | **Questions / probes** |
| --- | --- | --- | --- |
| **1** | Personal experience | Rapport building  General views and opinions of palliative care. | 1) Tell me please about your position / role   - How long have you been working in palliative care? - How did you decide to work in palliative care?   2) How changes/reforms in the past affected patients and PC workers? |
| **2** | Involvement of family caregivers into palliative care | To discover the current involvement and burden of family caregivers in the PC process | 1. To what extent family members are involved in caregiving in your hospice?  - Are all patients treated by family caregivers in your hospice? If they are not, how does it happen?  1. How would you evaluate the effect of caregiving on mental health of a family?  - If it is stressful, how do you help them to cope with stress?  1. Does the hospice staff provide training to family caregivers?  - If yes, can you describe me how and how long?  1. What are the different tasks/activities that family caregivers carry?   Do you think that some tasks are more appropriate for family member to do than for a professional?  Do you have experiences with some caregivers who carry out tasks that are not up to them ? For example ?   - If you encounter difficulties what are common problems you have with family caregivers? |
| **3** | Communication with family caregivers | Understand the quality of communication between doctors and family caregivers | 1. Do you try to involve more families into caregiving process? if yes, how…  - Is it easy to promote open communication ? If no .. What are the difficulties ? How do you manage this? - If yes, how do you seek to promote open communication, mutual support and teamwork?  1. Do patients and caregivers share their concerns, emotions and thoughts with your staff? 2. Is it possible to provide knowledge of palliative care to potential patients and caregivers? How do you do this ?  - Ok now with patients in need of palliative care from countryside, do you change something in your discourses? Perhaps communicate in a different way or adapt information? How do you communicate with patients in need of palliative care from countryside? |
| **4** | Identifying status quo in palliative care in Kazakhstan | Assess current quality and scope of palliative care services in the country | Now more general questions about the situation of palliative care in Kazakhstan:    1) How would you assess the quality of palliative care services in Kazakhstan?   - How would you assess the funding of palliative care in Kazakhstan? - How would you assess the role of the government and its support? - What are the common difficulties in this work? And in the everyday work? and at the hospice? - What should be improved? - At a large scale (government)? - In daily work in your hospice? - with the family caregivers (customs, uses) |
| **5** | Closing questions | Views and opinions | 1. How satisfied are you by your job? 2. Is there anything else you would like to share on the topic? |

**Interview guide for Administrators**

|  | **Theme** | **Aim** | **Questions / probes** |
| --- | --- | --- | --- |
| **1** | Status quo | Discover challenges of health administration of current state of PC | 1. Tell me please about your position / role 2. How long do you work in palliative care management? 3. How did you decide to work in PC? 4. Tell me please about your education/ training in PC? 5. Could you please describe your tasks and organization with PC practitioners/ hospice staff? 6. How do you collaborate / communicate with PC practitioners / hospices staff? 7. How do you manage the coordination of the work with physicians / nurses/ family caregivers?  - What is average salary of PC practitioners (doctors, nurses) - How many people work in PC in Kazakhstan? What is staff turnover in Kazakhstan? Its reasons and ways to reduce?  1. How trainings / educational programs and courses are organized for PC practitioners? If yes, can you please describe them? 2. How palliative care is funded in Kazakhstan? And usually elsewhere? |
| **2** | Development of palliative care in independent Kazakhstan | Discover which strategies were successful in the past and plans for the future | 1. Can you describe how PC has been developed since Kazakhstan became independent in 1991?  - What are the most significant milestones? - How it affected patients and PC workers?  1. How would you describe current state / quality of PC in Kazakhstan?  - What are achievements? - What are difficulties? - What remains to be improved?  1. Are you perhaps involved in some international discussions or meeting, congress of PC?  - How often do you participate in conferences/meetings/congresses? - You told me about international standards ( or first what the international standards ? ) - Is it possible to follow these standards ? why not |
| **3** | Identifying caregiver burden | To discover the current involvement and burden of family caregivers in the PC process | 1. How do you think the involvement of family caregivers affects palliative care?  - How they can help improve patients’ experience? - To what extent are they involved in the care? |
| **4** | Supplement | To identify important aspects in the provision of palliative care that have not been discussed yet. | 1. In your opinion, does the government provide enough contribution to make PC in Kazakshtan better? Why do you think so? 2. What are plans of your organization for the future?  - Vision and strategy  1. Can you describe the future of family caregiving in Kazakhstan? Either your hopes or fears for the future? (or both ) 2. Is there anything else you would like to discuss? |

**Interview guide for Nurses**

|  | **Theme** | **Aim** | **Questions / probes** |
| --- | --- | --- | --- |
| **1** | Personal experience | Rapport building  General views and opinions of palliative care. | 1. Tell me please about your position / role  - How long do you work in palliative care? - How did you decide to work in palliative care?  1. Tell me please about your education/ training in PC? 2. Could you please describe procedures that you provide? |
| **2** | Understand experiences of formal caregivers | Discover challenges of formal caregivers, e.g. nurses | 1. How would you describe your experience as a nurse providing care to patients with cancer? 2. How do you balance working in palliative care and your personal life?  - How satisfied are you by your job? - How do you cope with stress?  1. Tell me please more about your experience of working with family caregivers?  - Which procedures do you delegate to family caregivers? - Are they trained? If yes, by whom and what kind of training do they have? - How family caregivers affect your work and care outcomes? - Which procedures do you delegate to family caregivers? |
| **3** | Supplement | Identify difficulties that influence caregiving process and strategies for improvements | 1. Is there anything else you would like to discuss? |
