# Supplementary material for: Improving palliative care outcomes in remote and rural areas of LMICs through family caregivers: lessons from Kazakhstan
Source: Front Public Health. 2023 Aug 3;11:1186107. doi: 10.3389/fpubh.2023.1186107 (PMC10434554; doi:10.3389/fpubh.2023.1186107)
Supplement: Supplementary file 3 [file Table_3.DOCX]

| **Theme** | **Subtheme** | **Quote** |
| --- | --- | --- |
| 1) Lack of awareness about palliative care in the society. | Negative attitude to palliative care. | **Quote 1:** *"Also, our population is not even aware of the existence of hospices. Thousands of suffering families could have benefit from it, but they do not know what palliative care means. Those who know, have a negative attitude to it.... the word "hospice" is stigmatized".* (Administrator)  **Quote 2:** *"I am sure that people in rural areas have never even heard the word “hospice” in their lives and do not know what palliative care is, so care for terminal patients there lies solely on shoulders of family members".* (Administrator) |
|  | Conflicts with families. | **Quote 3:** *"Often we have to deal with patients and families who think that we treat cancer. Very often we are asked why we don’t do chemotherapy, surgery, or radiotherapy.... people are very often discontent about it.... we had so many conflicts with families because of this confusion".* (Physician)  **Quote 4:** “*People do not understand the meaning and importance of palliative care. You know… I see how families feel… as if they betrayed their loved one by bringing him here. Often, they angry at us … because we care, not cure”* (Physician) |
|  | Social events to promote palliative care. | **Quote 5:** *"It is hard for me to say but social ads, like posters, talks, or publicity campaigns could help. Sometimes we organize open days or volunteer days to raise people's awareness of palliative care. But all these efforts could be made in vain if there are no clear recommendations given to patients by their physicians, oncologists or polyclinics."* (Nurse)  **Quite 6: *“****Every year on World Hospice and Palliative Care Day we organize events dedicated to palliative care awareness. Many people participate, we walk the streets, hand out bulletins, conduct surveys and explain the meaning of palliative care to all.”* (Administrator) |
|  | Poor referral by oncologists. | **Quote 7:** *"Who diagnoses terminal cancer? Us? No!*  *Oncologists do that. And it is their responsibility to inform patients and their families that we, palliative care units and hospices, are ready to help 24/7. But often they don't do it and patients with terminal cancer go home. The same is true for other specialists, those who diagnose terminal stages of HIV, tuberculosis, stroke, and so on. They must inform and refer patients to us immediately after a terminal diagnosis."* (Physician)  **Quote 8:** *“One of the issues lies outside of the scope of palliative care, and it is how oncologists communicate to terminal patients about stopping cancer treatment, as it becomes useless or even harmful, and how they talk about transitioning to palliative care. I see that the problem is there, and I know it.”* (Administrator) |
| 2) Poor formal palliative care education. | Learning by trial and error. | **Quote 9:** *"The quality of palliative education is poor and hours not sufficient. Nurses start their practice in palliative care right after graduation without knowing its basics (...) To put it bluntly, they learn as they work with terminal patients by trial and error."* (Physician) |
|  | Lack of faculty members. | **Quote 10:** *“It is simple to introduce new courses, but*  *where are we going to find faculty? Palliative faculty. We do not have qualified teaching workforce, that is why we invite international experts and organize webinars, but it's still a drop in the ocean”.* (Physician) |
|  | Lack of training abroad. | **Quote 11:** "*I also take part in organizing international advanced trainings and courses for health professionals from Kazakhstan, but for all these years I haven't seen any single palliative care specialist sent for such trainings abroad".* (Administrator) |
|  | Experienced nurses teach new nurses. | **Quote 12:** *"Colleges do not teach us palliative care at all. Often, we hire nurses who have just graduated from college. We spend much time teaching them here. They learn a lot in the first months from older nurses. Then they quickly, (...) how to say, get the hang of it, and soon leave for another job in oncology or intensive care; And then, a new nurse takes a vacant position, and everything starts all over again".* (Nurse) |
|  | Ubiquitous training in palliative care for all. | **Quote 13:** “*Absolutely all physicians and nurses, regardless of specialty, and even us, family members, we all should have basic palliative care and pain management skills. This should be a priority in all settings: hospices, palliative units, big cities, and small villages”.* (Family caregiver).  **Quote 14:** “*We already have advanced postgraduate courses. But for broader coverage and availability, the introduction of palliative online courses could be an affordable and effective solution to the improve palliative literacy even in remote regions of our vast country”.* (Administrator) |
| 3) Absence of practical skills training for family caregivers. | Critical role of family caregivers. | **Quote 15:** *“Teaching families the principles of patient care is one of the most important needs in home and inpatient settings. Our palliative care system relies heavily on family caregivers, but it takes training and support to prepare families to the process of caregiving. To do that, we have to teach them, and make this knowledge and skills available to all”.* (Physician)  **Quote 16:** *“…I would say, they're the ones that hold everything together. They represent a critical pillar of palliative care which we must strengthen and support with all our might. And giving them access to courses and education is the first thing we must do in our setting.”* (Administrator) |
|  | Training for family caregivers. | **Quote 17**: *"Our nurses show them everything in detail: how to take care of a patient, how to prevent bedsores, how to change a diaper, how to install a colostomy bag. It really depends on family involvement. If a relative asks, we teach, if they don't, we can't make them come and take care."* (Physician) |
|  | Training for rural population. | **Quote 18**: *"Hospice patients represent just a small proportion of the total palliative needs in the country, while thousands of patients are hidden from our eyes because they are at home or live in rural areas. Reaching these families should be a priority when discussing family education because palliative illiteracy there is widespread."* (Nurse) |
|  | Online training courses for family caregivers. | **Quote 19**: “*It would be very helpful to have access to online educational resources, where I could get information about how to provide care to my mom at home, or at what stage I should seek hospice care; and most importantly, what is the outcome of palliative care”.* (Family caregiver)  **Quote 20**: “*It would be very helpful for us if families would have access to certain educational courses…. maybe online… where they are taught some palliative skills and given information in a clear and detailed way. Our nurses would complement it here by demonstrating some procedures”.* (Physician) |
| 4) Lack of mobile palliative care services for home-based care. | Home care after discharge. | **Quote 21:** *"It is very strange that after some time these patients are discharged; they are still terminally ill, they are not cured, why are they discharged as if they have already been cured? In this case, why are there no home-based care to continue palliative care until the end of the life?"* (Family caregiver)  **Quote 22:** *"We would really like to be supervised at home. Better at home than here. Home is familiar environment, psychologically it is better. It would be easier for all of us and my mother. I would prefer my mother to be monitored by a mobile service that visits us several times a week to give us advice and to be in touch with us at all times."* (Family caregiver) |
|  | Mobile teams are unavailable. | **Quote 23:** *“Many families prefer receiving palliative care at home.* *They might just wish dying at home, where their children are around them. But that doesn't mean we shouldn't take care of them. We must provide them with best home-based care possible, which is currently available only in some regions”.* (Nurse)  **Quote 24**: *"Our mobile team is a great help for hospices and cancer centers, it reduces their workload, its efficiency is not less than in the hospice. Our team visits around 8 patients every day and we receive very positive feedback from families. But as strange as it may sound, we have only one mobile palliative care team for a city of two million people, can you imagine that?"* (Physician) |
|  | Mobile teams as a supplement to family caregivers. | **Quote 25:** “*The availability of qualified and equipped mobile teams can be a very effective measure in conjunction with trained family members. For example, a mobile team visits patients, adjusts treatments, and stays in touch with a family member who is trained and prepared to provide palliative care at home”.* (Physician) |
| 5) Severe shortages of opioids to prevent pain suffering. | Short list of analgesics. | **Quote 26:** "*The list of opioid analgesics is very short: the weak opioid tramadol, short-acting injectable morphine, and the fentanyl patch. These are the only medicines we were given for severe pain management."* (Family caregiver) |
|  | Excessive control of opioids. | **Quote 27:** *“Primary reason of severe shortage of morphine in Kazakhstan is the very low volume of its imports. The overcontrol of opioids arises from the state's fear of its non-medical use and trafficking, which results in sufferings of the most vulnerable population. We are trying to reach out to both the Ministry of Health and the Ministry of the Interior. Oral morphine is still the gold standard of pain relief, and we don't have it at all.”* (Administrator) |
|  | Opiophobia among physicians. | **Quote 28: “***Physicians require us to return empty opioid ampoules before prescribing new ones. They must prescribe opioids to all patients in severe pain. Until we solve this problem, an increase of morphine imports makes no sense”.* (Family caregiver)  **Quote 29:** *"Before discussing shortage of opioids, we need to raise the issue of widespread opiophobia among physicians. Many patients in our country die in painful agony. Why? Because physicians are literally afraid to prescribe opioids, and when they do, they force patients to return old ampoules to get new dosages. Or even worse, to get a dose of opioids, the patient has to be admitted to hospice."* (Administrator) |
| 6) Funding that does not cover actual expenses. | Inability to cover costs. | **Quote 30:** *"Of course, there is not enough funding, I can say with certainty that the funding that we have today covers about 70% of the actual costs, which is already not bad. In addition, we rely on family caregivers, charity or sponsors."* (Physician)  **Quote 31:** *" Low funding also affects the range of medicines in our unit. We are forced to ask family caregivers to buy certain medicines, it is very embarrassing for us, and sometimes we buy medicines ourselves, out of our pocket."* (Nurse) |
|  | Out-of-pocket expenditures. | **Quote 32:** *"We have bought many consumables ourselves, and that, day after day, takes a lot of money. Especially now that I'm a student and my mother is no longer working, not only does she not get a salary, but we are now also spending 200 dollars a month on her treatment."* (Family caregiver)  **Quote 33:** *“When my husband was admitted, physicians asked me if he used an oxygen concentrator. He did not. Then I’ve bought him an oxygen concentrator for 700 dollars. I understand that our cancer centers do not have it because it is very expensive equipment.”* (Family caregiver) |
|  | Low salaries and high turnover. | **Quote 34:** *"Palliative nurses have a salary of around $250. Every month they fight for shifts to get a little more money. They often take more shifts than the Ministry of Labor allows. As a result, their productivity falls, and burnout increases. Moreover, our hospice has received fines for exposing nurses to this kind of burnout, and we have even less money left over. What is the bottom line? The nurses just leave the job."* (Administrator) |
| 7) Lack of the State support. | Importance of the State support. | **Quote 35:** "*The state should be a locomotive of palliative care development, and we, NGOs, sponsors, charity, we are like carriages, from behind we help the locomotive to move further. All these years palliative care in Kazakhstan has been developing independently, without a big role of the state, but we are about to exhaust these abilities, it's time for the state to come on stage and play its role."* (Administrator) |
|  | Frustration by the state support. | **Quote 36:** *"After all the difficult experiences I had with my mother, I got the feeling that government authorities are not concerned with these patients, they would rather focus on the strong and healthy. It's as if they have a consumerist attitude toward people; in other words, why should they help fund and assist these patients if they're going to die soon anyway?"* (Family caregiver) |
|  | Future of palliative care. | **Quote 37:** *"As an oncologist, I often attend oncology conferences and congresses, and I notice that palliative care is getting more attention in Kazakhstan. It seems to me that we still have everything ahead of us, I am sure the government will help, and we are just at the beginning of our way."* (Physician) |
|  | Collaboration with the State. | **Quote 38:** “*Better cooperation between palliative stakeholders and the state is the key to getting the state to support palliative care. We are already working on this. There were several meetings with the Minister of Health, and she has promised to support palliative care. The roadmap for the development of pediatric palliative care is being developed and we have a lot of work to do”.* (Administrator) |
